# p53 Dimers Associate with a Head-to-Tail Response Element to Repress Cyclin B Transcription

**DOI:** 10.1371/journal.pone.0042615

**Published:** 2012-08-08

**Authors:** Robert Lipski, Daniel J. Lippincott, Brittany C. Durden, Anne R. Kaplan, Hilary E. Keiser, Jung-Ho Park, Aime A. Levesque

**Affiliations:** Department of Biology, University of Hartford, West Hartford, Connecticut, United States of America; University of Illinois at Chicago, United States of America

## Abstract

DNA damage induced by the topoisomerase I inhibitor SN38 activates cell cycle checkpoints which promote cell cycle arrest. This arrest can be abrogated in p53-defective cells by the Chk1 inhibitor 7-hydroxystaurosporine (UCN-01). Previously, we compared p53 wild-type MCF10A cells with derivatives whose p53 function was inhibited by over-expression of the tetramerization domain (MCF10A/OD) or expression of shRNA against p53 (MCF10A/Δp53). Treatment of SN38-arrested MCF10A/OD cells with UCN-01 abrogated S, but not G2 arrest, while the MCF10A/Δp53 cells abrogated both S and G2 arrest. The MCF10A/OD cells had reduced levels of cyclin B, suggesting that tetramerization of p53 is not required for repression of cyclin B gene expression. In the present study, we analyzed p53 oligomerization status using glutaraldehyde cross-linking. Following SN38 treatment, MCF10A cells contained oligomeric forms of p53 with molecular weights approximating monomers, dimers, trimers, and tetramers. However, MCF10A/OD cells possessed only monomers and dimers suggesting that these complexes may be involved in repression of cyclin B. While genes transcriptionally activated by p53 contain a consensus sequence with elements repeated in a head-to-head orientation, the cyclin B promoter contains similar elements oriented head-to-tail. Chromatin immunoprecipitation (ChIP) assays revealed that p53 associates with this head-to-tail element in both MCF10A and MCF10A/OD. Electrophoretic mobility shift assays (EMSA) using a biotin-labeled probe containing the head-to-tail element showed a shift in mobility consistent with the molecular weight of tetramers and dimers in MCF10A nuclear extract, but only the dimer in MCF10A/OD nuclear extract. Taken together, these results suggest a novel mechanism whereby p53 dimers associate with the head-to-tail element to repress cyclin B transcription.

## Introduction

DNA damage activates cell cycle checkpoints that arrest cell cycle progression to facilitate repair. If the damage is irreparable, the cells undergo apoptosis. The tumor suppressor p53 plays an important role in both the cell cycle arrest and apoptosis in response to DNA damage by activating and repressing expression of a number of target genes (reviewed in [1]). Under normal circumstances, p53 is kept inactive by association with hDM2, an E3 ubiquitin ligase that targets p53 for degradation [2], [3]. Upon DNA damage, p53 is phosphorylated by checkpoint kinases such as ATM and ATR at serine 15, or Chk1 and Chk2 at serine 20 [4]–[7]. The phosphorylation leads to dissociation of hDM2 and stabilization of p53. The now activated p53 can both induce and repress expression of many genes involved in cell cycle regulation and apoptosis (reviewed in [8]–[10]).

Among the genes activated by p53 is hDM2, providing a negative regulatory feedback loop to control p53 activity [11]. p53 also activates transcription of target genes involved in cell cycle arrest, such as p21^ waf1^, a cyclin-dependent kinase (CDK) inhibitor that arrests cell cycle progression [12], 14-3-3σ, which sequesters the cyclin B-CDK1 complex outside the nucleus to prevent the onset of mitosis [13], [14], and GADD45, which dissociates cyclin B from CDK1 [15], [16]. p53 also represses transcription of cyclin B [17], [18], thereby providing a second level of protection against premature onset of mitosis in the presence of DNA damage.

**Figure 1:**
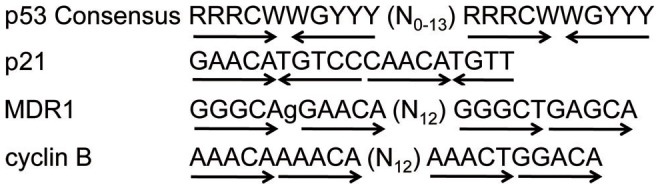
Comparison of head-to-head and head-to-tail p53 response elements. The consensus sequence of the standard p53 response element consists of two half-sites, each with quarter-sites oriented in a head-to-head manner (indicated by arrows). R =  purine, Y =  pyrimidine, W = A/T, and N = A/T/C/G. The p21 promoter contains a standard head-to-head p53 response element, while the MDR1 and cyclin B genes contain p53 response elements with head-to-tail orientations.

In order to activate transcription of target genes, p53 binds to a response element in the promoter of these genes. The standard p53 response element is a consensus sequence consisting of two repeats of head-to-head oriented half-sites of the sequence RRRCWWGYYY separated by 0–13 bp (figure 1, [19]). Each p53 response element whole-site can be bound by one p53 tetramer, with each half-site bound by one dimer [20]. One gene that contains this p53 response element is p21^waf1^ (figure 1, [21]), which contains two separate p53 response elements. Binding of p53 to the response elements in the p21^waf1^ promoter results in activation of p21^waf1^ transcription. The resulting increase in p21^waf1^ protein levels leads to loss of Cdk activity, and thus a block in cell cycle progression [22], [23].

The mechanism by which p53 represses expression of genes such as cyclin B is not well understood. p53 may regulate cyclin B gene expression through direct binding to a consensus sequence in the cyclin B promoter, through interaction with other transcription factors, or through a combination of these mechanisms (reviewed in [9]). The cyclin B promoter lacks the standard p53 response element, but instead contains a novel head-to-tail oriented response element at position -600 relative to the transcription start site (figure 1, [24]). A similar head-to-tail sequence was shown to be required for p53-mediated repression of the MDR1 gene, and reorientation to the head-to-head arrangement led to activation of MDR1 gene expression (figure 1, [24]). This same study also showed that p53 binds directly to this sequence; however, the orientation of this element makes it unlikely that a normal p53 tetramer could bind due to structural limitations [20]. The possibility that p53 binds to this element as a monomer or dimer remains open.

In our previous studies, we analyzed the effect of loss of p53 function in a p53 wild-type breast epithelial cell line, MCF10A [25]. When MCF10A cells were treated with the topoisomerase I inhibitor SN38, the cells arrested in the cell cycle, either in S or G2 depending on the concentration of drug. When arrested cells were treated with the Chk1 inhibitor 7-hydroxystaurosporine (UCN-01), the cells remained arrested in S phase. We generated two isogenic derivatives of the MCF10A cell line, one over-expressing the p53 tetramerization domain (MCF10A/OD), and the other expressing shRNA against p53 (MCF10A/Δp53). Over-expression of the tetramerization domain has previously been shown to prevent formation of p53 tetramers [26], while expression of the shRNA against p53 results in significantly reduced levels of p53 protein [25]. The MCF10A/Δp53 cells abrogated S and G2 arrest when treated with SN38 followed by UCN-01. These cells had low levels of p21^waf1^ and high levels of cyclin B, indicating that both the transcription induction and repression functions of p53 were lost. The same results (checkpoint abrogation, low levels of p21^waf1^, and high levels of cyclin B) have been observed in p53-defective breast cancer cells [27]. Surprisingly, when the MCF10A/OD cells were treated with SN38 followed by UCN-01, the cells abrogated S phase arrest, but not G2. These cells had reduced levels of p21^ waf1^ compared to controls, indicating that the transcription activation function of p53 was lost. However, the cells had low levels of cyclin B, indicating that the transcription repression function of p53 was maintained [25]. These results led us to propose that, unlike the activation of p21^ waf1^ expression, the p53-dependent repression of cyclin B expression does not involve tetramers.

In this study, we investigated the mechanism of p53-dependant repression of cyclin B transcription. Specifically, we used glutaraldehyde cross-linking to confirm the absence of tetramers and the presence of dimers in MCF10A/OD cells. We also determined whether p53 binds to the head-to-tail response element. Our results indicate that p53 dimers associate with this head-to-tail element, and that this association is sufficient for repression of cyclin B transcription.

## Results

**Figure 2:**
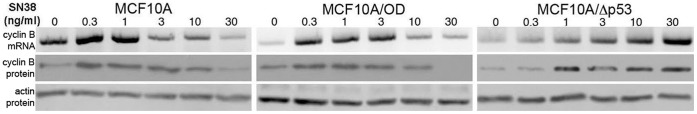
Determination of cyclin B mRNA and protein levels. MCF10A, MCF10A/OD, and MCF10A/Δp53 cells were incubated with 0–30 ng/ml SN38 for 24 hours. RNA was purified from cell lysates and relative levels of cyclin B mRNA were analyzed using RT-PCR followed by agarose gel electrophoresis. Cell lysates were also analyzed for expression of cyclin B and actin protein using SDS-PAGE followed by immunoblotting with cyclin B- or actin-specific antibodies.

**Figure 3:**
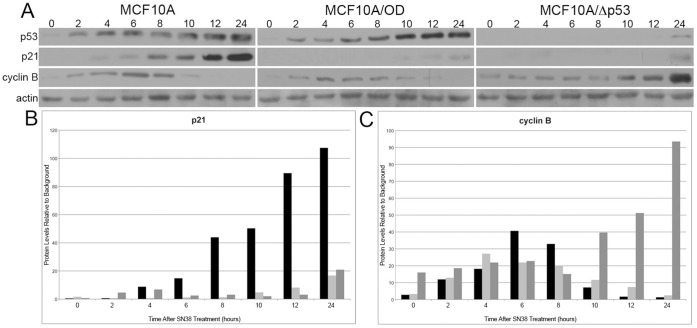
Timecourse of cyclin B and p21 expression following SN38 treatment. MCF10A, MCF10A/OD, and MCF10A/Δp53 cells were treated with 30 ng/ml SN38. **A**, Cells were harvested at the indicated time points and lysates were analyzed using SDS-PAGE followed by immunoblotting with the indicated antibodies. Band intensity of scanned immunoblots was measured using Image J software and the measurements were normalized to background. **B**, p21 and **C**, cyclin B levels were plotted for MCF10A (black bars), MCF10A/OD (light gray bars), and MCF10A/Δp53 (dark gray bars).

We have previously observed that treatment of MCF10A and MCF10A/OD cells with 0–30 ng/ml of SN38 results in a reduction of cyclin B protein levels at the highest concentrations, while the same treatment of MCF10A/Δp53 cells does not [25]. In order to confirm that this p53-dependent decrease in cyclin B expression is due to transcriptional repression, we analyzed cyclin B mRNA levels in these three cell lines in response to incubation with SN38. The mRNA levels in these cells correlated with protein levels (figure 2), indicating that the decrease in cyclin B protein levels represents a transcriptional event. We also compared the expression of cyclin B, p53 and p21 over 0–24 hours of treatment with 30 ng/ml of SN38 (figure 3). While p21 increased significantly in MCF10A cells beginning around 8 hours, there was negligible increase in p21 in the MCF10A/OD and MCF10A/Δp53 cells (figure 3A,B). The cyclin B protein increased transiently in MCF10A and MCF10A/OD cells at 4 to 6 hours before decreasing dramatically by 12 hours (figure 3A,C). In contrast, the cyclin B protein levels continued to increase dramatically through 24 hours in MCF10A/Δp53 (figure 3A,C). These results confirm our earlier observations that cyclin B is still repressed in cells over-expressing the tetramerization domain (MCF10A/OD), but not in cells lacking p53 (MCF10A/Δp53) [25].

**Figure 4:**
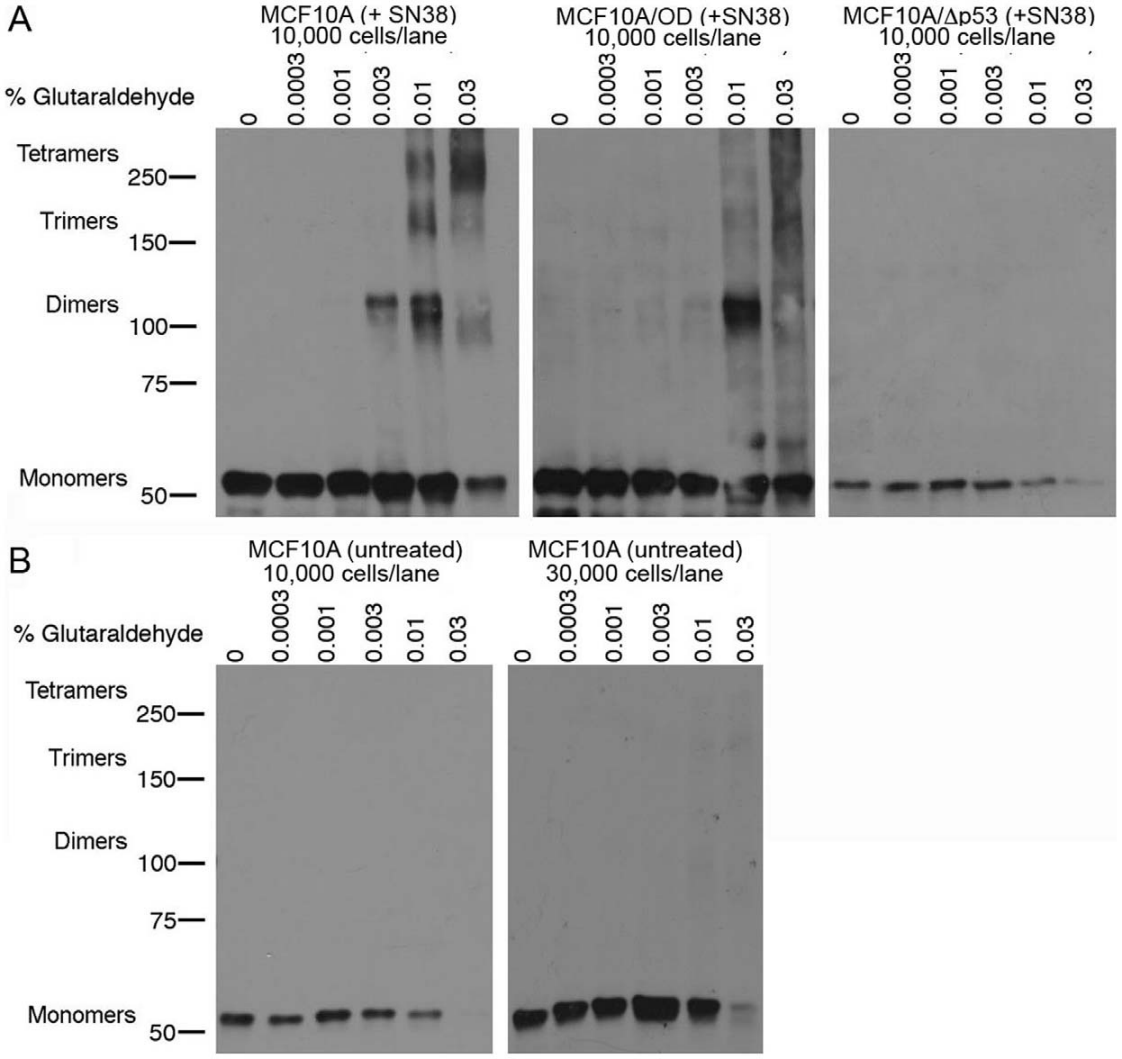
Determination of p53 oligomerization status using glutaraldehyde cross-linking. **A**, MCF10A, MCF10A/OD, and MCF10A/Δp53 and cells were incubated with 10 ng/ml SN38 (+SN38) for 24 hours or **B**, fresh media (untreated) for 24 hours. Cells were harvested in lysis buffer and incubated with the indicated concentrations of glutaraldehyde for 5 minutes. The cross-linked lysates were then analyzed by SDS-Page followed by immunoblotting with p53-specific antibodies. Cell lysates were loaded at 10,000 or 30,000 cells/lane as indicated.

In order to detect p53 oligomers, cell lysates were cross-linked with concentrations of glutaraldehyde ranging from 0% to 0.03% and then were analyzed by Western blotting. Similar concentrations have previously been used to observe p53 oligomerization [28], [29]. In MCF10A cells incubated with low concentrations of glutaraldehyde, monomers were abundant and some dimers were visible, but no tetramers were observed (figure 4A). At high concentrations of glutaraldehyde, primarily tetramers and trimers were seen, with only limited amounts of monomers and dimers. It should be noted that the oligomeric forms did not run at the expected molecular weights. For example, the tetramers appeared to run at a molecular weight higher than 250 kDa, while the predicted molecular weight would be 212 kDa. This is likely due to the fact that cross-linked proteins are non-linear and form a matrix of singularly or multiply cross-linked forms. These present a more complex structure that does not electrophorese according to predicted parameters. It is also noteworthy that the levels of p53 protein appear to decrease at higher concentrations of glutaraldehyde. This is not surprising, since glutaraldehyde likely blocks the epitope when present at a high enough concentration. The doublet bands seen for dimers (∼100 kDa) could represent different conformations of p53 dimers or perhaps one of the bands represents a heterodimer of p53 and another protein. These observations are damage-specific events that occur only in the presence of a DNA damaging agent (SN38). Untreated MCF10A cells lacked oligomers even when three times the number of cells was analyzed (figure 4B).

We also assessed the oligomerization status of p53 in MCF10A/OD cells. Presumably, the MCF10A/OD cells should not have tetramers because over-expression of the tetramerization domain would interfere with tetramerization [26]. However, dimerization should not be affected because it is mediated through a different domain [30]. We observed that the MCF10A/OD cells contained monomers and dimers, but lacked trimers and tetramers (figure 4A). Additionally, MCF10A/Δp53 cells possessed only low levels of monomers similar to basal levels seen in untreated MCF10A cells, and lacked higher molecular weight oligomers (figure 4). These observations suggest that p53 monomers or dimers could be required for repression of cyclin B expression, since the MCF10A/OD cells still repress cyclin B despite the absence of trimers and tetramers. These data offer strong support to our original hypothesis that tetramers are not involved in repression of cyclin B expression.

**Figure 5:**
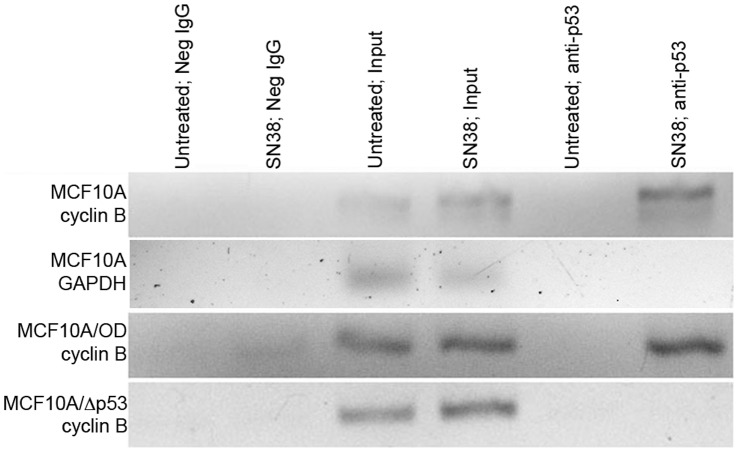
Analysis of p53 binding to head-to-tail response element using chromatin immunoprecipitation assays. MCF10A, MCF10A/OD, and MCF10A/Δp53 cells were incubated with either fresh media (untreated) or 10 ng/ml SN38 for 24 hours, followed by cross-linking in 1% formaldehyde. Chromatin was then purified, sonicated, and immunoprecipitated with anti-p53 antibody or negative IgG antibody. Unprecipitated chromatin was used as a positive control (input). DNA was isolated and amplified by PCR using primers flanking the head-to-tail response element in the cyclin B promoter or the RNA Polymerase II binding site from the GAPDH promoter. The amplified PCR fragments were analyzed by agarose gel electrophoresis.

We next assessed the association of p53 with the head-to-tail response element in the cyclin B promoter using chromatin immunoprecipitation (ChIP) assays. ChIP assays were performed using untreated cells as well as cells treated with SN38. Sonicated chromatin was immunoprecipitated with p53-specific antibodies or negative control mouse IgG. Unprecipitated sonicated chromatin was also used as the positive control input DNA. Chromatin samples were PCR amplified using primers flanking the head-to-tail element in the cyclin B promoter. For both the MCF10A and MCF10A/OD cells, a PCR product was present in the input samples as well as the SN38-treated p53 immunoprecipitate (figure 5). This indicates that p53 is bound to the head-to-tail element in the MCF10A and MCF10A/OD cells. Since the latter cells lack tetramers, this suggests that another form of p53 is bound to this element in these cells. The binding of p53 appears to be a damage specific response, since no PCR product was present in the untreated p53 immunoprecipitate. For the MCF10A/Δp53 cells, no PCR product was produced in the SN38-treated p53 immunoprecipitate. This is not surprising, since these cells contain p53 protein levels similar to that seen in untreated MCF10A cells, and lack oligomers (figures 3 and 4). To control for the presence of non-specific DNA in the p53 immunoprecipitate, the MCF10A samples were also amplified using primers flanking the RNA Polymerase II binding site in the glyceraldehyde-3-phosphate dehydrogenase (GAPDH) promoter, and PCR products were only produced in the input sample and not in the p53 immunoprecipitate (figure 5).

**Figure 6:**
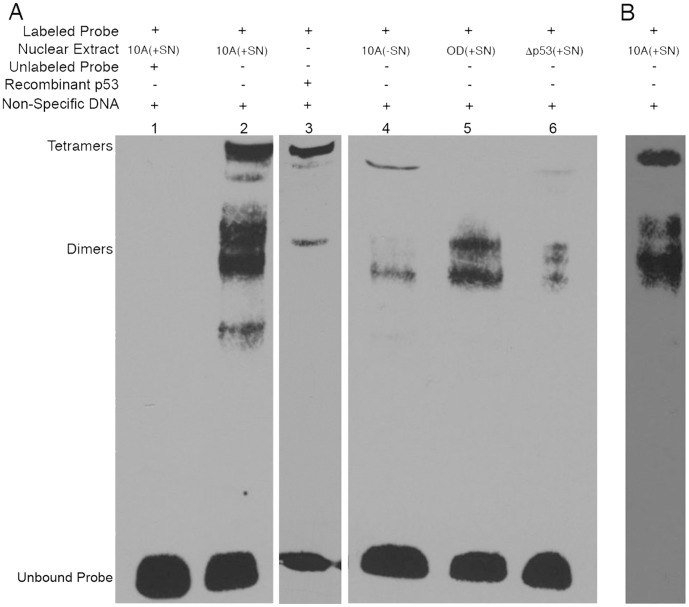
Analysis of p53 binding to head-to-tail response element in cyclin B promoter using electrophoretic mobility shift assays. **A**, MCF10A, MCF10A/OD, and MCF10A/Δp53 cell lines were incubated with 10 ng/ml SN38 for 24 hours (+SN) or were left untreated (–SN). Binding reactions were prepared by incubating nuclear extracts or recombinant p53 with a biotin-labeled probe corresponding to the head-to-tail response element in the cyclin B promoter, in the presence (+) or absence (−) of a 200-fold molar excess of specific DNA (unlabeled probe). All binding reactions were carried out in the presence of a 100-fold molar excess of unlabeled non-specific DNA. Complexes were separated on 4% native polyacrylamide gel electrophoresis, transferred to positively charged nylon membrane, and visualized using a streptavidin-horse radish peroxidase conjugate. **B**, A parallel gel was transferred to nitrocellulose membrane and immunoblotted with p53-specific antibodies.

We used electrophoretic mobility shift assays (EMSA) to determine whether p53 can bind to the head-to-tail element in the cyclin B promoter as a monomer or dimer. This technique has previously been used to observe association of different oligomeric forms of p53 to specific DNA sequences [31]. Incubation of biotin-labeled probe corresponding to the head-to-tail element from the cyclin B promoter with nuclear extract from SN38-treated MCF10A cells resulted in multiple shifted bands corresponding in molecular weight to different oligomeric forms of p53 (figure 6A, lane 2). The dimers appeared as a doublet, similar to the case seen in glutaraldehyde cross-linking (figure 4A). Co-incubation with a 200-fold molar excess of the unlabeled specific DNA probe resulted in loss of these shifted bands (figure 6A, lane 1), confirming that the shift was specific to the head-to-tail response element. Additionally, a 100-fold molar excess of unlabeled non-specific DNA was included in all binding reactions to eliminate the possibility of non-specific DNA binding (figure 6). The presence of p53 in these shifted bands was confirmed by simultaneously immunoblotting this sample for p53 (figure 6B).

The occurrence of two bands around the molecular weight of a dimer suggests that at least one of these bands may consist of a complex of p53 and another protein as opposed to a p53 dimer. In order to address this possibility, the binding reaction was repeated using recombinant p53 protein rather than MCF10A nuclear extract. This resulted in two bands, one similar in molecular weight to the tetrameric shifted band seen in the MCF10A nuclear extract, and another similar in molecular weight to the higher of the two dimeric bands (figure 6A, lane 3). This suggests that the higher band represents the true p53 dimers, while the lower dimer band may represent a heterodimer of p53 and another lower molecular weight protein.

The electrophoretic shift was a damage-specific event, as nuclear extract from untreated MCF10A cells had significantly reduced levels of all shifted bands (figure 6A, lane 4), which resembled the effects of incubation with nuclear extract derived from SN38-treated MCF10A/Δp53 cells (figure 6A, lane 6). These results lend additional support to the conclusion that p53 is present in the shifted complex, since cells known to have reduced levels of p53 protein also have reduced levels of the shifted bands. Interestingly, nuclear extract derived from SN38-treated MCF10A/OD cells resulted in shifted bands corresponding to the dimeric molecular weights, but not the tetrameric form (figure 6A, lane 5). Taken together, these results suggest that p53 binds to the head-to-tail response element as a dimer.

## Discussion

Cyclin B plays an important role in preventing entry into mitosis in the face of DNA damage. p53 has been shown to down-regulate cyclin B expression and thereby prevent entry into mitosis [32] while over-expression of cyclin B causes premature entry into mitosis [33]. Additionally, cyclin B over-expression has been associated with high tumor grade in squamous cell carcinomas of the head and neck [34], and thus may represent a prognostic biomarker for some types of cancer. Our previous studies have shown that cells over-expressing the p53 tetramerization domain are insensitive to checkpoint inhibitors because they still repress cyclin B [25]. The results of the present study suggest a novel mechanism of p53-mediated gene regulation in which p53 dimers mediate cyclin B gene repression through association with a head-to-tail response element.

The glutaraldehyde cross-linking studies revealed that cells over-expressing the p53 tetramerization domain lack tetramers, but still possess dimers and monomers (figure 4A). Since these cells still repress cyclin B expression in a manner similar to the case seen in p53-wildtype MCF10A cells (figures 2 and 3), this result suggests that the p53-dependent repression of cyclin B could be mediated by monomers or dimers. The results of the ChIP assays support the conclusion that p53 associates with the head-to-tail element as a monomer or dimer, because p53 is still associated with the element even in the MCF10A/OD cells that lack tetramers (figure 5). The EMSA results enable us to rule out the possibility that p53 monomers are involved in repression of cyclin B transcription because there was no shifted band present at molecular weights approximating monomers when EMSA was performed using MCF10A/OD nuclear extract (figure 6, lane 5).

The fact that recombinant p53 resulted in shifted bands approximating dimers and tetramers suggests that p53 binds to this sequence directly (figure 6, lane 3). p53 has previously been shown to bind directly to a similar head-to-tail sequence in the MDR1 promoter [24]. However, this does not necessarily mean that p53 binding alone is sufficient to repress cyclin B expression. The complete picture of p53-mediated cyclin B repression remains to be elucidated (reviewed in [9]). Some groups have reported that p53 represses cyclin B gene expression by interacting with other promoter bound transcription factors, such as Myc/Max, NF-Y, and SP1. One report suggests that p53-mediated repression of cyclin B expression involves interaction of p53 with a single Myc/Max-regulated E-box element located at position −189 in the cyclin B promoter [35]. Others have reported that p53 interacts with NF-Y, which has three binding sites in the cyclin B promoter located at positions −80, −48, and one downstream of the transcription start site [36], [37]. Lastly, it has been reported that p53 mediates repression of cyclin B expression by interacting with SP1, which has two recognition sequences in the cyclin B promoter at positions -259 and −140 [18]. Some of these reports directly contradict one another, but the possibility that p53 regulates cyclin B expression through a combination of one or more of these mechanisms remains open.

Wild-type p53 protein alone is not sufficient to mediate activation or repression of target gene expression. We have previously observed that some p53 wild-type tumor cell lines (HCT116 and MCF7) are sensitive to checkpoint inhibitors [38]. These cells still activate p53 in response to treatment with SN38, but induction of p21^waf1^ and repression of cyclin B are attenuated [38]. These results suggest that these cells possess defects in the p53 pathway that affect both the activation and repression functions. Several co-activators and co-repressors have been implicated in mediating p53 transcriptional activity (reviewed in [39], [40]). Thus, it is likely that some cells with wild-type p53 have defects in one or more of these co-factors required for activation and repression.

The canonical p53 response element found in genes whose expression is activated by p53 consists of two repeats of the head-to-head oriented half-sites, each with the sequence of 5′-RRRCWWGYYY-3′, separated by a spacer of 0–13 bp (figure 1, [19]). It has been shown that the specific sequence of the core “CWWG” as well as the length of the spacer are critical to achieving maximal activation (reviewed in [39]). Importantly, the core sequence of CATG and spacer length of less than 3 bp are optimal in order to achieve activation [39]. When p53 binds to response elements in DNA, it does so as a “dimer of dimers”, that is, one dimer binds to each half-site, which leads to a conformational change of both the p53 protein and the DNA in that region of the promoter [30], [40]. It is not known whether tetramer formation occurs before binding of individual dimers to the two half-sites or after, although at least one study suggests that the latter is true [41].

In the case of the cyclin B promoter, it is unlikely that a tetramer would bind to the head-to-tail response element with high affinity because the spacer length of 12 bp is non-optimal, the core sequence is CAAA instead of CATG, and the orientation of the binding sites would make it difficult for two dimers to bind to both sequences and to each other to form a tetramer. Two alternative models are possible. In the first, two dimers bind to each of the two half-sites in the cyclin B promoter, but do not associate with each other to form a tetramer. However, one would predict that if this were the case, EMSA using MCF10A/OD nuclear extract would have resulted in a shifted band corresponding to the molecular weight of tetramers, which was not the case (figure 6, lane 5). A more likely model is that only one dimer can associate with the element at a time, possibly by binding to both half-sites simultaneously. Alternatively, binding of a dimer to only one half-site may be sufficient to achieve repression. Surprisingly, the EMSA results suggest that tetramers are able to associate with the head-to-tail response element (figure 6 lane 2). However, it cannot be determined from our data whether tetramers have the ability to repress expression of cyclin B, just that they associate with the head-to-tail element. It is clear from the MCF10A/OD data that in the absence of tetramers, dimers are sufficient to associate with the head-to-tail response element and repress cyclin B expression.

Although a functional role of p53 dimers in gene repression has not previously been reported, there are studies that suggest that tetramerization of p53 is not necessarily essential for gene activation. It has been observed that binding of p53 to promoters containing only half-sites of the p53 response element is sufficient for activation of gene expression, although activation from half-sites is reduced compared to activation from whole-sites [42]–[44]. Since p53 dimers typically bind to each half-site, it is possible that p53 dimers are mediating gene activation under these circumstances.

The results of this study suggest a novel mechanism of p53-mediated gene regulation and shed light on the mechanism of p53-mediated gene repression. We have shown that p53 associates with a head-to-tail response element as a dimer and that this association is sufficient to suppress cyclin B expression in cells lacking tetramers. Future studies will be aimed at further dissecting the pathway of cyclin B expression by identifying co-factors required for p53 mediated repression as well as identifying the specific base pairs within the head-to-tail response element essential for p53 binding.

## Materials and Methods

### Chemicals

SN38, the active metabolite of the topoisomerase I inhibitor irinotecan, was provided by Pfizer Global (Kalamazoo, MI). For all treatments with SN38, cells were plated and after 24 hours were given fresh media supplemented with drug, or fresh media alone in the case of untreated cells. All SN38 treatments were performed for 24 hours unless otherwise indicated. For all experiments where responses of different cell lines to treatment were compared, the same number of cells was always utilized.

### Cell Culture

The p53 wild-type breast cell line MCF10A (American Type Culture Collection, Manassas, VA, USA) was maintained in DMEM/F12 media supplemented with 10% fetal bovine serum, penicillin (100 U/ml) streptomycin (100 mg/ml) and fungizone (0.25 mg/ml), 8 mg/ml insulin, 20 ng/ml epidermal growth factor and 500 ng/ml hydrocortisone. The MCF10A/OD cell lines over-expresses amino acids 276–369 of p53, which corresponds to the tetramerization domain. These cells were previously referred to as MCF10A/AL [25]. The MCF10A/Δp53 cell line expresses shRNA against p53. The synthesis of the MCF10A/OD and MCF10A/Δp53 cell lines was previously described [25]. These cells were maintained in the MCF10A media supplemented with 500 µg/ml G418, which was removed before all experimental treatments.

### Immunoblotting

For immunoblot analysis of SN38 dose-response and timecourse samples, cells were rinsed with phosphate buffered saline, and then lysed by direct addition of Laemmli sample buffer. Samples were immediately boiled for 5 min and stored at −20**°**C. Proteins were separated by SDS–PAGE using 8% gels (10% for p21 blots) and transferred onto polyvinylidene difluoride membranes. Membranes were blocked with 5% non-fat milk in Tris-buffered saline, 0.1% Tween 20, and then probed with the appropriate antibody overnight at 4**°**C [cyclin B (GNS1), p53 (DO-1), and p21 (C-19) from Santa Cruz Biotechnology, Santa Cruz, CA; β-actin from Cell Signaling Technology, Beverly, MA]. Subsequently, membranes were washed in Tris buffered saline, 0.1% Tween 20, and incubated with secondary antibody conjugated to horseradish peroxidase (BioRad, Hercules, CA). Proteins were visualized using LumiGlo chemiluminescence substrate (KPL, Inc., Gaithersburg, MD). For all experiments where multiple cell lines were analyzed for the same protein, the same exposure times were used.

In order to measure changes in protein levels, immunoblot films were scanned, and the files were subsequently analyzed using Image J software (National Institutes of Health, USA). The intensity of each band was measured by drawing an oval shaped box around the band. The same area was measured for each band for all antibodies and cell lines, and values were normalized against a background region of equal area on the same film for each set of samples.

### Glutaraldehyde Cross-linking

In order to analyze the oligomerization status of p53, glutaraldehyde cross-linking assays were performed using a modification of a previously reported method [45]. Cells were treated with 10 ng/ml of SN38 for 24 hours, after which the cells were rinsed in phosphate buffered saline. Cells were lysed in lysis buffer (10 mM Tris pH 7.6, 140 mM NaCl, 0.5% NP40) supplemented with protease inhibitor and phosphatase inhibitor cocktails (Sigma Aldrich, St. Louis, MO). Glutaraldehyde solutions of varying concentration (0–0.3% final concentration) were added to each lysate, and samples were incubated for 5 min at room temperature. Laemmli sample buffer was added to each sample; samples were then boiled for 5 min and analyzed by SDS-PAGE using 6% polyacrylamide gels followed by immunoblot. Cells were loaded on the gel at 10,000 or 30,000 cells per lane.

### RNA Purification, cDNA Synthesis, and Polymerase Chain Reaction

The transcript level of cyclin B1 was assessed by PCR. Total RNA was isolated using Qiagen’s RNeasy Mini kit. Synthesis of cDNA was performed for 30 minutes at 55°C with 1 µg of RNA using Superscript III One-Step RT-PCR System with Platinum Taq (Invitrogen, Carlsbad, CA). The primers used were as follows: B1-forward 5′ AAGAGCTTTAAACTTTGGTCTGGG 3′; B1-reverse 5′ CTTTGTAATGCCTTGATTTACCATG 3′ [46]. Amplification was performed for 35 cycles with 15 sec at 94°, 30 sec at 55° and 1 min 68°C. This generated a single band of 317 bp when analyzed by electrophoresis using 2% agarose gels.

### Chromatin Immunoprecipitation Assays

Chromatin immunoprecipitation (ChIP) assays were used in order to observe binding of p53 to specific target sequences. ChIP assays were performed using Active Motif’s ChIP-IT kit following manufacturers instructions. Briefly, cells were cross-linked with 1% formaldehyde, and chromatin was purified and sonicated. A portion of the sonicated chromatin was set aside as the input sample. The sonicated chromatin was pre-cleared using salmon sperm DNA/protein G agarose, followed by immunoprecipitation with p53-specific antibodies (DO-1, Santa Cruz) or negative control mouse IgG (Active Motif, Carlsbad, CA). The cross-links were reversed by heating to 65°C, and DNA was purified from both the immunoprecipitated samples and the input samples.

Polymerase chain reaction (PCR) of the purified DNA samples was used to amplify the head-to-tail element in the cyclin B promoter. The PCR primers utilized were as follows: cycBforward 5′CCTGATTTTCCCATGAGAGG3′; cycBreverse 5′GGATCACACATTAGCAACGGG3′. As a control, the samples were analyzed by PCR using primers to amplify the RNA Polymerase II site from the glyceraldehyde-3-phosphate dehydrogenase (GAPDH) promoter. The primers were as follows: GAPDHforward 5′TACTAGCGGTTTTACGGGCG3′; GAPDHreverse 5′TCGAACAGGAGGAGCAGAGAGCGA3′. Amplification was performed for 30 cycles with 20 sec at 94°C, 30 sec at 55.5°C, and 30 sec at 72°C. This generated in a single band of 300 bp (for cyclin B) or 166 bp (for GAPDH) when analyzed by electrophoresis using 2% agarose gels.

### Electrophoretic Mobility Shift Assays

DNA probes corresponding to the head-to-tail element in the cyclin B promoter were generated by first obtaining single stranded oligos 42 nts in length with the following sequences: sense, 5′AAACAAACAAAACAATTGGCCTTGGGAAACTGGACAATCTTG3′; antisense, 5′CAAGATTGTCCAGTTTCCCAAGGCCAATTGTTTTGTTTGTTT3′. These oligos were labeled at the 3′ end using the Biotin 3′ End DNA Labeling Kit (Thermo Scientific, Rockford, IL) following manufacturers instructions. The labeled oligos were then annealed by mixing equal molar amounts of the two single-stranded oligos, heating to 95°C for 5 minutes, followed by ramp cooling to 25°C over a period of 45 minutes.

Nuclear extracts were prepared using the NE-PER Nuclear and Cytoplasmic Extraction Reagents (Thermo Scientific) following the manufacturer’s instructions. Electrophoretic mobility shift assays (EMSA) were performed using the LightShift Chemiluminescent EMSA Kit (Thermo Scientific) following the manufacturer’s instructions. All binding reactions were carried out in the presence of 2 pmol of the non-specific DNA Poly (dI•dC) (Thermo Scientific), 20 fmol of biotin-labeled DNA probe, 2.5% glycerol, 0.05% NP40, 1.5 mM MgCl_2_, and 1 mM EDTA. For reactions carried out in the presence of nuclear extract, 9 µg total protein was utilized. Recombinant p53 protein (Enzo Life Sciences, Farmingdale, NY) was used at a final concentration of 0.15 µM in place of nuclear extract in some binding reactions. Additionally, 4 pmol of unlabeled probe was added to some binding reactions as specific competitor DNA.

Complexes were separated by electrophoresis on a native 4% polyacrylamide gel in 0.5X TBE and transferred to a positively charged nylon membrane. The electrophoretic mobility of the complexes was detected using streptavidin-horse radish peroxidase (HRP) and a chemiluminescent substrate (Thermo Scientific).

## References

[1] Meek DW (2004) The p53 response to DNA damage. DNA Repair (Amst) 3(8–9): 1049–1056. 15279792 10.1016/j.dnarep.2004.03.027

[2] Haupt Y, Maya R, Kazaz A, Oren M (1997) Mdm2 promotes the rapid degradation of p53. Nature 387(6630): 296–299. 9153395 10.1038/387296a0

[3] Kubbutat MH, Jones SN, Vousden KH (1997) Regulation of p53 stability by Mdm2. Nature 387(6630): 299–303. 9153396 10.1038/387299a0

[4] Banin S, Moyal L, Shieh S, Taya Y, Anderson CW, et al (1998) Enhanced phosphorylation of p53 by ATM in response to DNA damage. Science 281(5383): 1674–1677. 9733514 10.1126/science.281.5383.1674

[5] Canman CE, Lim DS, Cimprich KA, Taya Y, Tamai K, et al (1998) Activation of the ATM kinase by ionizing radiation and phosphorylation of p53. Science 281(5383): 1677–1679. 9733515 10.1126/science.281.5383.1677

[6] Hirao A, Kong YY, Matsuoka S, Wakeham A, Ruland J, et al (2000) DNA damage-induced activation of p53 by the checkpoint kinase Chk2. Science 287(5459): 1824–1827. 10710310 10.1126/science.287.5459.1824

[7] Shieh SY, Ahn J, Tamai K, Taya Y, Prives C (2000) The human homologs of checkpoint kinases Chk1 and Cds1 (Chk2) phosphorylate p53 at multiple DNA damage-inducible sites. Genes Dev 14(3): 289–300. 10673501 PMC316358

[8] Taylor WR, Stark GR (2001) Regulation of the G2/M transition by p53. Oncogene 20(15): 1803–1815. 11313928 10.1038/sj.onc.1204252

[9] Ho J, Benchimol S (2003) Transcriptional repression mediated by the p53 tumour suppressor. Cell Death Differ 10(4): 404–408. 12719716 10.1038/sj.cdd.4401191

[10] Yu J, Zhang L (2005) The transcriptional targets of p53 in apoptosis control. Biochem Biophys Res Commun 331(3): 851–858. 15865941 10.1016/j.bbrc.2005.03.189

[11] Wu X, Bayle JH, Olson D, Levine AJ (1993) The p53-mdm-2 autoregulatory feedback loop. Genes Dev 7(7A): 1126–1132. 8319905 10.1101/gad.7.7a.1126

[12] Hengstschlager M, Braun K, Soucek T, Miloloza A, Hengstschlager-Ottnad E (1999) Cyclin-dependent kinases at the G1-S transition of the mammalian cell cycle. Mutat Res 436(1): 1–9. 9878675 10.1016/s1383-5742(98)00022-2

[13] Chan TA, Hermeking H, Lengauer C, Kinzler KW, Vogelstein B (1999) 14–3-3Sigma is required to prevent mitotic catastrophe after DNA damage. Nature 401(6753): 616–620. 10524633 10.1038/44188

[14] Laronga C, Yang HY, Neal C, Lee MH (2000) Association of the cyclin-dependent kinases and 14–3-3 sigma negatively regulates cell cycle progression. J Biol Chem 275(30): 23106–23112. 10767298 10.1074/jbc.M905616199

[15] Kastan MB, Zhan Q, el-Deiry WS, Carrier F, Jacks T, et al (1992) A mammalian cell cycle checkpoint pathway utilizing p53 and GADD45 is defective in ataxia-telangiectasia. Cell 71(4): 587–597. 1423616 10.1016/0092-8674(92)90593-2

[16] Zhan Q, Antinore MJ, Wang XW, Carrier F, Smith ML, et al (1999) Association with Cdc2 and inhibition of Cdc2/Cyclin B1 kinase activity by the p53-regulated protein Gadd45. Oncogene 18(18): 2892–2900. 10362260 10.1038/sj.onc.1202667

[17] Taylor WR, DePrimo SE, Agarwal A, Agarwal ML, Schonthal AH, et al (1999) Mechanisms of G2 arrest in response to overexpression of p53. Mol Biol Cell 10(11): 3607–3622. 10564259 10.1091/mbc.10.11.3607 PMC25646

[18] Innocente SA, Lee JM (2005) p53 is a NF-Y- and p21-independent, Sp1-dependent repressor of cyclin B1 transcription. FEBS Lett 579(5): 1001–1007. 15710382 10.1016/j.febslet.2004.12.073

[19] el-Deiry WS, Kern SE, Pietenpol JA, Kinzler KW, Vogelstein B (1992) Definition of a consensus binding site for p53. Nat Genet 1(1): 45–49. 1301998 10.1038/ng0492-45

[20] McLure KG, Lee PW (1998) How p53 binds DNA as a tetramer. EMBO J 17(12): 3342–3350. 9628871 10.1093/emboj/17.12.3342 PMC1170672

[21] Chin PL, Momand J, Pfeifer GP (1997) In vivo evidence for binding of p53 to consensus binding sites in the p21 and GADD45 genes in response to ionizing radiation. Oncogene 15(1): 87–99. 9233781 10.1038/sj.onc.1201161

[22] Xiong Y, Hannon GJ, Zhang H, Casso D, Kobayashi R, et al (1993) P21 is a universal inhibitor of cyclin kinases. Nature 366(6456): 701–704. 8259214 10.1038/366701a0

[23] el-Deiry WS, Harper JW, O’Connor PM, Velculescu VE, Canman CE, et al (1994) WAF1/CIP1 is induced in p53-mediated G1 arrest and apoptosis. Cancer Res 54(5): 1169–1174. 8118801

[24] Johnson RA, Ince TA, Scotto KW (2001) Transcriptional repression by p53 through direct binding to a novel DNA element. J Biol Chem 276(29): 27716–27720. 11350951 10.1074/jbc.C100121200

[25] Levesque AA, Kohn EA, Bresnick E, Eastman A (2005) Distinct roles for p53 transactivation and repression in preventing UCN-01-mediated abrogation of DNA damage-induced arrest at S and G2 cell cycle checkpoints. Oncogene 24(23): 3786–3796. 15782134 10.1038/sj.onc.1208451

[26] Ossovskaya VS, Mazo IA, Chernov MV, Chernova OB, Strezoska Z, et al (1996) Use of genetic suppressor elements to dissect distinct biological effects of separate p53 domains. Proc Natl Acad Sci U S A 93(19): 10309–10314. 8816796 10.1073/pnas.93.19.10309 PMC38380

[27] Kohn EA, Ruth ND, Brown MK, Livingstone M, Eastman A (2002) Abrogation of the S phase DNA damage checkpoint results in S phase progression or premature mitosis depending on the concentration of 7-hydroxystaurosporine and the kinetics of Cdc25C activation. J Biol Chem 277(29): 26553–26564. 11953432 10.1074/jbc.M202040200

[28] Stenger JE, Mayr GA, Mann K, Tegtmeyer P (1992) Formation of stable p53 homotetramers and multiples of tetramers. Mol Carcinog 5(2): 102–106. 1554407 10.1002/mc.2940050204

[29] Itahana Y, Ke H, Zhang Y (2009) p53 oligomerization is essential for its C-terminal lysine acetylation. J Biol Chem 284(8): 5158–5164. 19106109 10.1074/jbc.M805696200 PMC2643511

[30] Kitayner M, Rozenberg H, Kessler N, Rabinovich D, Shaulov L, et al (2006) Structural basis of DNA recognition by p53 tetramers. Mol Cell 22(6): 741–753. 16793544 10.1016/j.molcel.2006.05.015

[31] Hanson S, Kim E, Deppert W (2005) Redox factor 1 (ref-1) enhances specific DNA binding of p53 by promoting p53 tetramerization. Oncogene 24(9) 1641–1647: 10.1038/sj.onc.1208351. 10.1038/sj.onc.1208351 15674341

[32] Innocente SA, Abrahamson JL, Cogswell JP, Lee JM (1999) p53 regulates a G2 checkpoint through cyclin B1. Proc Natl Acad Sci U S A 96(5): 2147–2152. 10051609 10.1073/pnas.96.5.2147 PMC26751

[33] Tam SW, Belinsky GS, Schlegel R (1995) Premature expression of cyclin B sensitizes human HT1080 cells to caffeine-induced premature mitosis. J Cell Biochem 59(3): 339–349. 8567752 10.1002/jcb.240590306

[34] Hoffmann TK, Trellakis S, Okulicz K, Schuler P, Greve J, et al (2011) Cyclin B1 expression and p53 status in squamous cell carcinomas of the head and neck. Anticancer Res 31(10): 3151–3157. 21965721 PMC3721303

[35] Yin XY, Grove L, Datta NS, Katula K, Long MW, et al (2001) Inverse regulation of cyclin B1 by c-myc and p53 and induction of tetraploidy by cyclin B1 overexpression. Cancer Res 61(17): 6487–6493. 11522645

[36] Manni I, Mazzaro G, Gurtner A, Mantovani R, Haugwitz U, et al (2001) NF-Y mediates the transcriptional inhibition of the cyclin B1, cyclin B2, and cdc25C promoters upon induced G2 arrest. J Biol Chem 276(8): 5570–5576. 11096075 10.1074/jbc.M006052200

[37] Imbriano C, Gurtner A, Cocchiarella F, Di Agostino S, Basile V, et al (2005) Direct p53 transcriptional repression: In vivo analysis of CCAAT-containing G2/M promoters. Mol Cell Biol 25(9): 3737–3751. 15831478 10.1128/MCB.25.9.3737-3751.2005 PMC1084283

[38] Levesque AA, Fanous AA, Poh A, Eastman A (2008) Defective p53 signaling in p53 wild-type tumors attenuates p21waf1 induction and cyclin B repression rendering them sensitive to Chk1 inhibitors that abrogate DNA damage-induced S and G2 arrest. Mol Cancer Ther 7(2): 252–262. 18281511 10.1158/1535-7163.MCT-07-2066

[39] Menendez D, Inga A, Resnick MA (2009) The expanding universe of p53 targets. Nat Rev Cancer 9(10): 724–737. 19776742 10.1038/nrc2730

[40] Ho WC, Fitzgerald MX, Marmorstein R (2006) Structure of the p53 core domain dimer bound to DNA. J Biol Chem 281(29): 20494–20502. 16717092 10.1074/jbc.M603634200

[41] Weinberg RL, Veprintsev DB, Fersht AR (2004) Cooperative binding of tetrameric p53 to DNA. J Mol Biol 341(5): 1145–1159. 15321712 10.1016/j.jmb.2004.06.071

[42] Menendez D, Krysiak O, Inga A, Krysiak B, Resnick MA, et al (2006) A SNP in the flt-1 promoter integrates the VEGF system into the p53 transcriptional network. Proc Natl Acad Sci U S A 103(5): 1406–1411. 16432214 10.1073/pnas.0508103103 PMC1360546

[43] Menendez D, Inga A, Snipe J, Krysiak O, Schonfelder G, et al (2007) A single-nucleotide polymorphism in a half-binding site creates p53 and estrogen receptor control of vascular endothelial growth factor receptor 1. Mol Cell Biol 27(7): 2590–2600. 17242190 10.1128/MCB.01742-06 PMC1899907

[44] Yan J, Menendez D, Yang XP, Resnick MA, Jetten AM (2009) A regulatory loop composed of RAP80-HDM2-p53 provides RAP80-enhanced p53 degradation by HDM2 in response to DNA damage. J Biol Chem 284(29): 19280–19289. 19433585 10.1074/jbc.M109.013102 PMC2740553

[45] Atz J, Wagner P, Roemer K (2000) Function, oligomerization, and conformation of tumor-associated p53 proteins with mutated C-terminus. J Cell Biochem 76(4): 572–584. 10653977 10.1002/(sici)1097-4644(20000315)76:4<572::aid-jcb6>3.0.co;2-6

[46] Krause K, Wasner M, Reinhard W, Haugwitz U, Dohna CL, et al (2000) The tumour suppressor protein p53 can repress transcription of cyclin B. Nucleic Acids Res. 28(22): 4410–4418. 10.1093/nar/28.22.4410 PMC113869 11071927

